# The High-Risk Human Papillomavirus E6 Oncogene Exacerbates the Negative Effect of Tryptophan Starvation on the Development of *Chlamydia trachomatis*

**DOI:** 10.1371/journal.pone.0163174

**Published:** 2016-09-22

**Authors:** Shardulendra P. Sherchand, Joyce A. Ibana, Arnold H. Zea, Alison J. Quayle, Ashok Aiyar

**Affiliations:** 1 Department of Microbiology, Immunology & Parasitology, LSU Health Sciences Center, 1901 Perdido Street, New Orleans, Louisiana, 70112, United States of America; 2 Institute of Biology, University of the Philippines, Diliman, Quezon City, Philippines, PH, 1101; Institut Pasteur, FRANCE

## Abstract

*Chlamydia trachomatis* is an obligate intracellular pathogen that requires specific essential nutrients from the host cell, one of which is the amino acid tryptophan. In this context interferon gamma (IFNγ) is the major host protective cytokine against chlamydial infections because it induces the expression of the host enzyme, indoleamine 2,3-dioxygenase 1, that degrades tryptophan, thereby restricting bacterial replication. The mechanism by which IFNγ acts has been dissected *in vitro* using epithelial cell-lines such as HeLa, HEp-2, or the primary-like endocervical cell-line A2EN. All these cell-lines express the high-risk human papillomavirus oncogenes E6 & E7. While screening cell-lines to identify those suitable for *C*. *trachomatis* co-infections with other genital pathogens, we unexpectedly found that tryptophan starvation did not completely block chlamydial development in cell-lines that were HR-HPV negative, such as C33A and 293. Therefore, we tested the hypothesis that HR-HPV oncogenes modulate the effect of tryptophan starvation on chlamydial development by comparing chlamydial development in HeLa and C33A cell-lines that were both derived from cervical carcinomas. Our results indicate that during tryptophan depletion, unlike HeLa, C33A cells generate sufficient intracellular tryptophan via proteasomal activity to permit *C*. *trachomatis* replication. By generating stable derivatives of C33A that expressed HPV16 E6, E7 or E6 & E7, we found that E6 expression alone was sufficient to convert C33A cells to behave like HeLa during tryptophan starvation. The reduced tryptophan levels in HeLa cells have a biological consequence; akin to the previously described effect of IFNγ, tryptophan starvation protects *C*. *trachomatis* from clearance by doxycycline in HeLa but not C33A cells. Curiously, when compared to the known *Homo sapiens* proteome, the representation of tryptophan in the HR-HPV E6 & E6AP degradome is substantially lower, possibly providing a mechanism that underlies the lowered intracellular free tryptophan levels in E6-expressing cells during starvation.

## Introduction

*Chlamydia trachomatis* is an obligate intracellular bacterium with a biphasic development in which it alternates between an infectious extracellular elementary body (EB), and an intracellular metabolically active but non-infectious reticulate body (RB) [1, 2]. Distinct *C*. *trachomatis* serovars are associated with ocular and genital infections of humans, with serovars D through K being tropic for columnar epithelial cells of the urogenital tract [3]. The ocular serovars of *C*. *trachomatis* are the leading infectious cause of blindness, and if left untreated, genital infections in women can result in severe consequences to their reproductive health as well as to neonatal well-being. After entry, EBs differentiate into RBs within a host-derived lipid vesicle termed a chlamydial inclusion. As the bacterium’s life-cycle proceeds, RBs replicate via binary fission within this inclusion, then re-differentiate into EBs that can infect bystander cells. This biphasic life-cycle with an obligate intracellular form has driven chlamydial evolution, such that the bacterium has lost the capacity to synthesize several essential nutrients *de novo* [4, 5]. Pertinent to the studies described here, all *C*. *trachomatis* isolates lack a complete pathway to synthesize tryptophan *de novo*, which renders *C*. *trachomatis* sensitive to growth restriction by the effects of the host cytokine interferon gamma (IFNγ). IFNγ induces the host enzyme indoleamine 2,3-dioxygenase that catabolizes intracellular tryptophan to kynurenine, thereby limiting the availability of this essential amino acid during chlamydial development [6, 7]. Under these conditions, chlamydial development is subject to an alternative program resulting in the formation of inclusions with aberrant RBs (ABs) that do not proceed to yield infectious EBs as long as tryptophan remains limiting [8–12]. While prolonged tryptophan starvation is bactericidal, tryptophan deprivation initially induces a bacteriostatic persistent state during which time re-provision of tryptophan can restore normal development.

Although all chlamydial isolates lack an intact tryptophan biosynthetic pathway, there is a critical distinction between ocular and genital serovars in the last step of this pathway, specifically, the capacity to synthesize tryptophan via indole salvage [13, 14]. This capacity is strictly conserved in every genital but no ocular isolate (*ibid*). Elegant studies, conducted *in vitro*, have shown that the addition of indole to *C*. *trachomatis*-infected cells exposed to IFNγ, restores normal development of all genital but no ocular clinical isolates [13, 14]. We, and others, have hypothesized that this conservation indicates the availability of indole in the genital compartment, most likely through the metabolic activities of the prevalent cervico-vaginal microbiome [15]. Consistent with this hypothesis, we have recently reported the isolation of indole-producing anaerobic bacteria from vaginal samples [15]. The findings described in this report resulted from control experiments conducted to identify an optimal human cell-line in which this hypothesis could be tested.

Previous studies examining chlamydial amino acid utilization have revealed cell-line specific effects of tryptophan deprivation [16]. Tryptophan was discovered to be advantageous but not essential for the development of *C*. *trachomatis* in McCoy cells [17]. In contrast, tryptophan-free media profoundly disrupts the growth of *C*. *trachomatis* development in HeLa cells (or the HeLa subclone, HeLa 229) [9]. Disparate effects of tryptophan starvation on chlamydial growth have also been observed within the same cell-line depending on its state of polarization [16]. Therefore, as a prelude to our co-culture studies, we screened multiple adherent cell-lines to identify those amenable for use in media lacking tryptophan. These cell-lines included HeLa, A2EN, C33A, and 293. We found tryptophan deprivation to dramatically reduce chlamydial development in HeLa and A2EN cells, both of which express the E6 and E7 oncogenes of high-risk human papillomavirus (HR-HPV). Surprisingly, tryptophan deprivation had a markedly smaller effect on chlamydial development in C33A and 293 cells, both of which are HR-HPV negative. We have dissected the reason for this difference by comparing HeLa and C33A cells, both of which are derived from human cervical cancers.

Our results indicate that intracellular levels of free tryptophan during starvation differ strikingly between HeLa and C33A cells, with the latter having substantially higher levels of free intracellular tryptophan during starvation. Two experimental observations permit us to ascribe this difference to proteasome function: a) Inhibition of proteasomes in C33A cells using MG132 blocks chlamydial development during tryptophan starvation; and b) Activating immunoproteasomes within HeLa cells restores chlamydial development during IFNγ-dependent tryptophan depletion. Despite presumably large genetic differences between HeLa and C33A cells, we demonstrate that the ectopic expression of the HPV16 E6 oncogene is sufficient to confer a HeLa-like phenotype to C33A cells during tryptophan starvation. Expression of HPV16 E6 in C33A cells reduces free intracellular tryptophan cells during tryptophan starvation and concurrently imposes a block to chlamydial development. Previous studies have shown that exposure to IFNγ reduces the effect of the antibiotic doxycycline on chlamydial development in HeLa cells [18]. Our findings indicate this observation to correlate with the amount of free intracellular tryptophan during starvation, such that doxycycline is more effective against chlamydial development when intracellular tryptophan stores are larger.

Finally, we report a bioinformatics/statistical analysis to explore why the expression of HR-HPV E6 might alter the size of the intracellular tryptophan pool during starvation. A detailed compositional analysis of proteins targeted for degradation by E6, or its partner ubiquitin ligase E6AP, reveal them to be remarkably, and significantly, poorer in tryptophan when compared to the entire known human proteome (Human genome project release GRCh38).

## Experimental Procedures

### Preparation of complete and drop-out media, and routine propagation of cell-lines

Dulbecco’s modified Eagle’s Medium (DMEM) was prepared by the formulation of Dulbecco, with the exceptions that phenol red was omitted and tryptophan was added at a final concentration of 4 mg/L (instead of 16 mg/L). The pH of different media batches ranged from 7.0–7.3. Complete Media was prepared by the addition of 10% 3X dialyzed fetal bovine serum (FBS) to DMEM. Batches of Complete Media were evaluated by measuring the growth of 293 cells over 72 hours. Tryptophan-free or arginine-free drop-out media were prepared by omitting tryptophan or arginine during media preparation. Trp-Free Media and Arg-Free Media were prepared by the addition of 10% 3X dialyzed FBS to the respective drop-out media. Trp-Free and Arg-Free media were evaluated by measuring the survival of 293 cells using MTS assays over 48 hours. Fetal bovine serum was obtained from Gemini Biosciences. The serum used for drop-out media was subjected to three rounds of dialysis against a 100-fold excess of PBS. The tryptophan concentration in drop-out media + 10% 3X dialyzed FBS was 80 μg/L as evaluated by reverse-phase HPLC. For routine propagation, HeLa cells were grown in DMEM + 10% FBS; C33A cells were grown in DMEM:F12 (1:1) + 5% FBS; 293 cells were grown in DMEM:F12 + 7% FBS; and A2EN cells were grown in DMEM + 5% FBS. Under these conditions, HeLa, C33A and 293 cells doubled every 20–22 hours. A2EN cells doubled every 30–36 hours. During routine propagation, cells were grown in the presence of penicillin (50 I.U/mL) and streptomycin (50 μg/mL).

### *C*. *trachomatis* infections, growth post-infection, and evaluation of IFU recovery

Cells were plated 24 hours prior to infection in antibiotic-free DMEM + 10% FBS. Cells were infected using *C*. *trachomatis* serovar D (D/UW-3/CX) at an m.o.i of 5, unless otherwise stated. HeLa, C33A, and A2EN infections were performed in SPG by centrifugation as described previously [19, 20]. 293 infections were infected by gently rocking at 4°C for an hour. After infection, SPG was replaced with Complete Media, Trp-Free Media or Arg-Free Media. IFU recovery for all growth conditions was quantified using HeLa cells grown under routine propagation conditions.

### Immunofluorescence staining for chlamydial inclusions and microscopy

Cells grown on coverslips were stained with the Merifluor anti-chlamydial LPS conjugated to FITC was used to stain *C*. *trachomatis* as described previously, and counterstained using Hoechst 33342 (1:3000) dilution for two minutes [19]. Stained cells were visualized during a Zeiss Axiovision AX10 microscope with a 63X oil-immersion objective (numerical aperture, 1.4). Z-stacks containing 20, 250 nm, optical sections were deconvolved using the Landweber positively constrained deconvolution algorithm as described previously [21]. Maximum intensity Z-projections are shown.

### Immunoblotting for IDO1, Ubiquitin, β-actin, eIF2α and p-eIF2α

Immunoblots were conducted as described previously [19, 20]. The following primary antibodies were used: 1) IDO1 –Millipore catalog 05–840; 2) Ubiquitin–a rabbit polyclonal antibody against ubiquitin kindly provided by Dr. Arthur Haas (Department of Biochemistry, LSUHSC); 3) β-actin–Sigma-Aldrich catalog A1978; 4) eIF2α –Pierce catalog MA1-079; and 5) p-eIF2α –Pierce catalog MA5-15133. This antibody detects eIF2α phosphorylated on serine 51.

### Doxycycline treatment and tryptophan starvation

Doxycycline was added at a final concentration of 1 μg/mL to the indicated samples. Cycloheximide was added at 1 μg/mL during recovery in complete media.

### MG132 and IFΝγ exposure

MG132 was obtained from Selleckchem (catalog S2619), and IFNγ was obtained from Peprotech (catalog 300–02). MG132 was solubilized in DMSO, which was used as the vehicle control. A titration was performed to determine the highest concentration of MG132 that did not compromise cell viability for 48 hours, and this concentration (0.3 μM) was used in experiments as indicated. MG132, or IFNγ at the indicated concentrations, was added immediately post-infection.

### Intracellular tryptophan measurement

Cell-lines were plated in Complete Media at 50% confluency. 24 hours later, this media was replaced with Complete Media or Trp-Free Media. 20 hours later, 10^8^ cells were harvested in PBS + 2 mM EDTA, counted using a Beckman Z1 Coulter counter, and their viability was evaluated by staining with trypan-blue or eosin-yellow. Control experiments were conducted in parallel with smaller numbers of cells to evaluate cell-health by Annexin V / propidium iodide (PI) staining. Cells grown in bulk were discarded unless their viability was >90%, with <5% staining positive for Annexin V or double-positive for Annexin V and PI. The difficulty in growing this large numbers of cells with the same batch of media and dialyzed serum, using the strict cutoff parameters for viability/health described here, permitted these measurements to only be conducted a single time. Cells were pelleted, washed in PBS, resuspended in 0.5 mL of 1.5 M HClO_4_ and vortexed. After this, 0.25 mL of 2M K_2_CO_3_ was added. Vortexed samples were pelleted for 5 minutes at 10000 x g, following which supernatants filtered through a 0.2 μm filter were used for RP-HPLC analysis performed as described previously [19]. A tryptophan standard curve was prepared in a HClO_4_/K_2_CO_3_ solution containing a range of concentrations from 1–100 μM. Tryptophan was detected using absorbance at 280 nm. The limit of tryptophan detection was 5 μM.

### Construction of C33A cell-lines expressing HPV16 E6, E7, and E6+E7

The pLXSN retroviral vector, and its derivatives expressing HPV16 E6, E6+E7, and E7 were prepared in gp293 cells using protocols published previously [22, 23]. Vector was purified using a 20% sucrose cushion and used immediately. Transduced cells were split 1:100 post-exposure to vector and selected using G418 (600 μg/mL). Under these conditions, cells transduced with a control-GFP vector that cannot confer G418 resistance died within 120 hours. Colonies were pooled after 14 days of selection. Viral gene expression was confirmed using RT-PCR [24].

### Proteome composition analysis

A FASTA format file containing the known and predicted human proteome (Human Genome Release GRCh38) was downloaded from NCBI. Mario Stenke’s Perl script *splitMfasta*.*pl* was used to split this file into ~73000 individual FASTA files. The Bash script, (S1 Text) was used to sequentially determine the amino acid composition of each protein, while appending the output into a tab-delimited file compatible with Excel. The GNU utility, *grep*, was used to eliminate all predicted proteins from the output file leaving only proteins known to exist by transcript analysis. This file is detailed in S1 Spreadsheet. All isoforms of a given protein were included in the compositional analysis. Literature searches were used to identify proteins degraded by HR-HPV E6 or E6AP. This list of proteins, with their composition, is detailed in S2 Spreadsheet. Citations revealing degradation for each protein by E6 or E6AP are indicated by a PMID adjacent to the identity of that protein. All isoforms of a given protein were included in the degradome compositional analysis, unless E6 or E6AP were shown to degrade just a specific isoform.

### Statistical analysis

All experiments were conducted at least three times. The mean and standard deviations was calculated using Microsoft Excel. MSTAT was used to perform statistical comparisons using the Wilcoxon rank sum test.

## Results

### The effect of tryptophan starvation on the development of *Chlamydia trachomatis* is cell-line dependent

**Fig 1:**
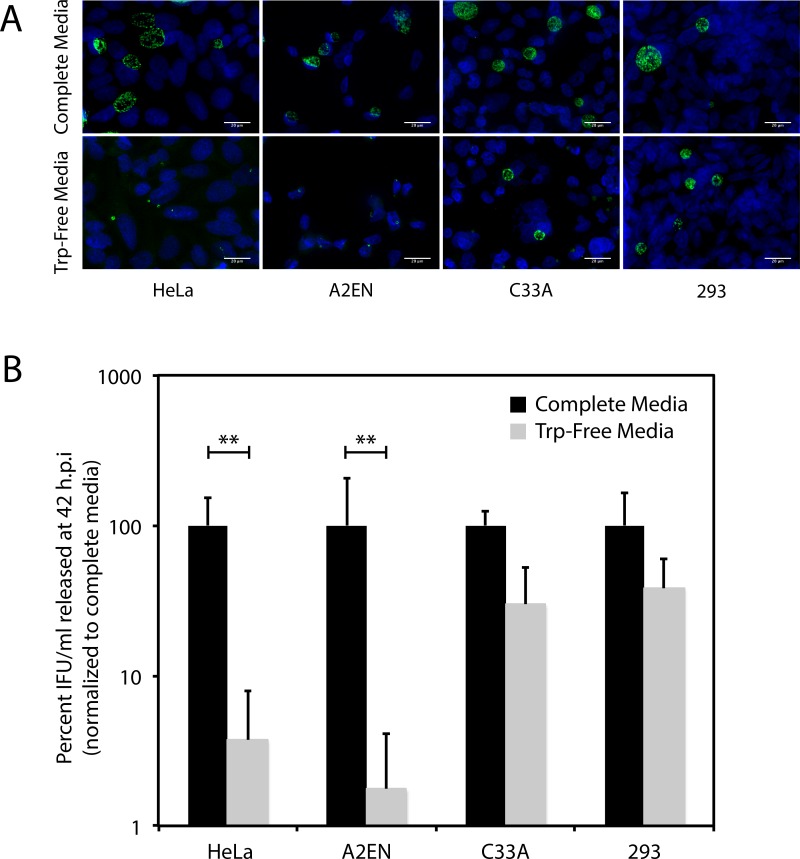
Cell-line dependent differences in the effect of tryptophan depletion on *C*. *trachomatis* development. Cells were infected with *C*. *trachomatis* at an m.o.i of 5 as described in the methods. After infection cells were maintained in complete media or tryptophan-free media. After 42 h.p.i. cells are either stained by immunofluorescence for chlamydial LPS using a FITC-conjugated antibody, or harvested to quantify IFU recovery by infection of HeLa cells. A) Chlamydial inclusions (green) formed in HeLa, A2EN, C33A and 293 cells 42 h.p.i. Cells were counterstained with Hoechst dye. Scale bar indicates 20 μm. B) IFU/mL recovered after 42 h.p.i. in HeLa, A2EN, C33A and 293 cells as evaluated by infection of HeLa cells. The bars represents mean and standard deviation from three independent experiments (** indicates P < 0.01 by the Wilcoxon rank sum test).

Because tryptophan is an essential amino acid for both *C*. *trachomatis* and its human host cells, we anticipated that the developmental cycle of *C*. *trachomatis* would be impeded to a similar extent in a variety of human epithelial cell-lines grown in media lacking tryptophan (Trp-Free Media) prepared as described in experimental procedures. This was tested by comparing the growth of *C*. *trachomatis* in Complete Media and Trp-Free Media using the following human epithelial cell-lines, whose origin and characteristics are described in Table 1: 1) HeLa; 2) A2EN; 3) C33A; and 4) 293 [25–29]. Cells were infected with *C*. *trachomatis* at an m.o.i of 5 as described in the experimental procedures. After infection, cells were incubated in Trp-Free Media (<80 ng/mL tryptophan) or Complete Media (4 μg/mL tryptophan), for 42 hours post-infection (h.p.i.) after which they were stained using FITC-conjugated anti-chlamydial LPS antibody or harvested to evaluate recovery of infectious units (IFU). The results of these analyses are shown in Fig 1. While the number and size of chlamydial inclusions were dramatically reduced in HeLa and A2EN cells grown in Trp-Free Media (Fig 1A), the effect of tryptophan starvation on inclusion number/size was much smaller in C33A and 293 cells (*ibid*). Consistent with this, IFU recovery at 42 h.p.i. was reduced by ~2-logs in HeLa and A2EN cells, but only by 50–70% in C33A and 293 cells (Fig 1B). *C*. *trachomatis* replicates to differing efficiency in these four cell-lines (detailed data for C33A and HeLa is shown in S1 Fig), therefore IFU recovery in Trp-Free media for each cell line is shown as a percent of the recovery for that cell line in Complete Media. In complete media, the IFUs/mL recovered at 42 h.p.i were: a) HeLa is ~1.6x10^6^/ml, b) C33A is ~0.5x10^6^/ml, c) A2EN is ~1.7x10^6^/ml, and d) 293 is ~2x10^6^/ml.

**Table 1:** Characteristics of the cell lines used in this study.

Cell Line	ATCC#	Tissue Origin	Known Viral Status	Reference
HeLa	CCL-2	Cervical Cancer	HPV 18 positive	[28]
C33A	HTB-31	Cervical Cancer	HPV negative	[25, 29]
A2EN	-	Endocervical epithelium	HPV16 E6/E7 transformed	[26]
293	CRL-1573	Human embryonic kidney	Adenovirus type 5 transformed	[27]

### During tryptophan starvation, HeLa cells have reduced levels of free intracellular tryptophan relative to C33A cells

The observations above are most easily explained if the levels of intracellular tryptophan differ between cell-lines during tryptophan starvation. Therefore, we measured the intracellular free tryptophan pool for uninfected cells grown for 20 hours in Complete Media or Trp-Free Media. This analysis, whose outcome is indicated in Table 2, was conducted using HeLa and C33A because both these cell-lines are derived from cervical cancers, have similar doubling times, but differ strikingly in chlamydial growth phenotypes during tryptophan starvation. As shown in Table 2, 20 hours after tryptophan withdrawal, free tryptophan levels in HeLa cells decreased by approximately 75%, contrasted to a 55% decrease observed in C33A cells, correlating with the reduced chlamydial development observed in HeLa cells.

**Table 2:** Intracellular free tryptophan concentrations in HeLa and C33A cells grown in Complete Media or after 20 Hours in Trp-Free Media.

Media Conditions	HeLa	C33A
Complete Media (Trp = 4 mg/L)	48.4 ± 7.1 nmoles^1^	61.5 ± 6.8 nmoles
Trp-Free Media (Trp = 80 μg/L)	11.7 ± 5.9 nmoles	26.3 ± 7.7 nmoles
Percent decrease in Trp-Free Media	76%	57%

^1^Tryptophan concentrations were evaluated using extracts from 10^8^ cells using RP-HPLC.

**Fig 2:**
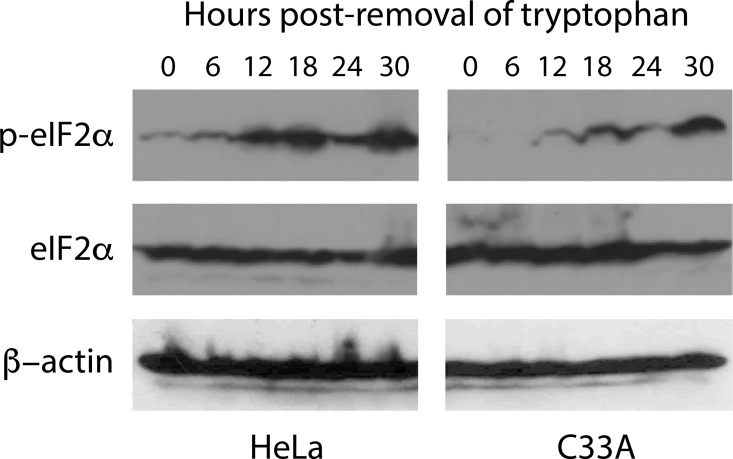
Temporal evaluation of eIF2α phosphorylation in HeLa and C33A grown in tryptophan-free media. HeLa and C33A cells were plated in complete media, which was replaced with tryptophan-free media 24 hours post-plating. Cells were harvested every 6 hours and used to make extracts that were evaluated by immunoblot using antibodies against eIF2α or eIF2α phosphorylated on serine 51 (p-eIF2α). Immunoblots against β-actin were performed as a loading control. Similar results were obtained from three independent experiments.

During amino acid starvation, protein synthesis in eukaryotes is blocked by phosphorylation of the eukaryotic translation initiation factor alpha (eIF2α) by the general control nonderepressible 2 kinase (GCN2 kinase) [30–32]. Therefore, we evaluated the phosphorylation status of eIF2α in HeLa and C33A cells as a function of time of exposure to Trp-Free Media. This analysis, shown in Fig 2, revealed eIF2α to be phosphorylated earlier in HeLa cells relative to C33A cells. In HeLa cells, eIF2α phosphorylation was observed immediately after tryptophan withdrawal, and maximal phosphorylation was observed by 12 hours post-withdrawal. In contrast, eIF2α phosphorylation was not observed in C33A cells until 12 hours post-withdrawal of tryptophan, with maximal phosphorylation of eIF2α being observed at 24–30 hours post-withdrawal. Thus, direct measurement of free intracellular tryptophan levels, and eIF2α phosphorylation kinetics, are consistent with each other and correlate with differences observed in chlamydial inclusion development and IFU release from HeLa and C33A cells during tryptophan starvation.

### The efficacy of doxycycline against *C*. *trachomatis* during tryptophan starvation is cell-line dependent

Previous studies using HeLa cells have demonstrated that the efficacy of doxycycline against *C*. *trachomatis* is impaired when the chlamydial developmental cycle has been arrested using IFNγ [18]. The reduced efficacy of doxycycline could occur for at least two reasons: 1) IFNγ might induce host proteins that attenuate the effect of doxycycline. For example, IFNγ induces the multidrug resistance 1 (MDR1) pump [33, 34], which could potentially secrete intracellular doxycycline thereby protecting replicating chlamydiae; or 2) Alternatively, by inducing the catabolism of tryptophan, IFNγ might reduce bacterial translation and therefore decrease the available targets for doxycycline. The distinction between these two mechanisms is of relevance while considering the development of anti-chlamydial vaccines that rely on the generation of a strong host IFNγ response, especially if the latter were to induce host genes that confer resistance to antibiotics. On the other hand, no such concerns arise if IFNγ protected against doxycycline by reducing the already diminished levels of intracellular tryptophan seen in HeLa cells. The dramatically different outcome of tryptophan starvation on *C*. *trachomatis* IFU release in C33A and HeLa cells led us to postulate that growth in Trp-Free Media could distinguish between these two possible mechanisms by which IFNγ might act to protect chlamydiae against doxycycline.

**Fig 3:**
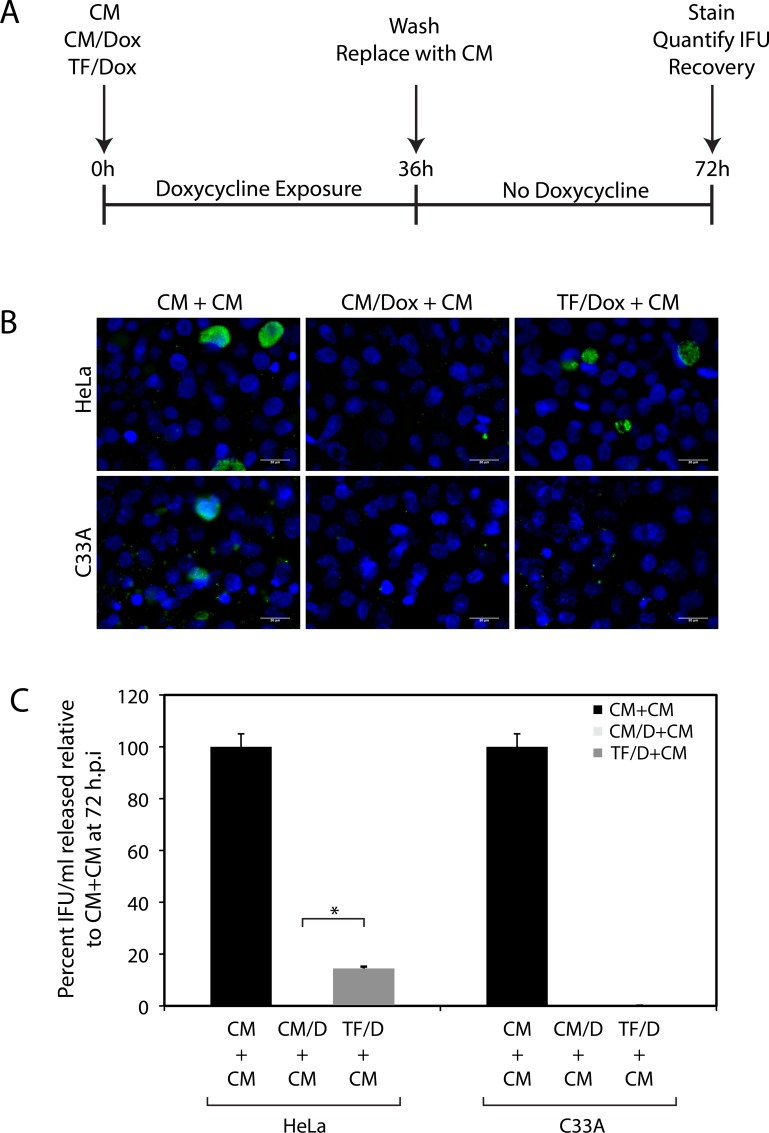
The efficacy of doxycycline treatment against *C*. *trachomatis* during tryptophan starvation is cell-line dependent. A) Schematic depiction of the experimental protocol. HeLa or C33A cells were infected with *C*. *trachomatis*. Post-infection, cells were maintained in complete media (CM), complete media with 1 μg/mL doxycycline (CM/Dox), or tryptophan-free media with 1 μg/mL doxycycline (TF/Dox). Infected cells were exposed to doxycycline for 36 hours, following which the media was replaced with complete media. After an additional 36 hours of growth in complete media, infected cells were evaluated for inclusion formation and IFU recovery. B) Immunofluorescence for chlamydial LPS to detect inclusions in infected HeLa and C33A cells under the three conditions described in “A”, i.e. CM + CM, CM/Dox + CM, and TF/Dox + CM. Cells were counterstained with Hoechst dye, and a scale bar of 20 μm is indicated. C) IFU/mL recovered under the three conditions indicated in “A” as a percent of the IFU/mL recovered under the CM + CM condition. IFU recovery was quantified by infection of HeLa cells. The bars represent the mean and standard deviation from three independent experiments. Significantly higher IFUs were recovered when infected HeLa cells were initially grown in the TF/Dox condition relative to the CM/Dox condition (* indicates p < 0.05 by the Wilcoxon rank sum test).

We hypothesized that *C*. *trachomatis* would be more susceptible to doxycycline exposure in C33A cells relative to HeLa cells during growth in Trp-Free Media, as the former contain higher levels of free intracellular tryptophan under these conditions, thus permitting higher levels of bacterial translation and providing targets for doxycycline. This hypothesis was tested as depicted schematically in Fig 3A. After infection at an m.o.i of 5, cells were grown for 24 hours in Complete Media (CM), complete media + 1 μg/mL doxycycline (CM+D), or Trp-Free Media + 1 μg/mL doxycycline (TF+D). *C*. *trachomatis* growth was evaluated by IFU recovery after an additional 36 hours of growth in doxycycline-free Complete Media. As shown in Fig 3B, tryptophan starvation protected *C*. *trachomatis* against doxycycline to a greater extent in HeLa cells relative to C33A cells. This difference is highly reminiscent of the previous observation that reducing intracellular tryptophan levels in HeLa cells by IFNγ exposure decreases *C*. *trachomatis* susceptibility to doxycycline. Therefore, although IFNγ has been shown to induce host genes, such as MDR1, which potentially may interfere doxycycline function/availability, IFNγ’s protective effect on replicating chlamydiae against doxycycline results from its induction of tryptophan catabolism.

### Proteasome activity is essential to support chlamydial development in C33A cells during tryptophan starvation

**Fig 4:**
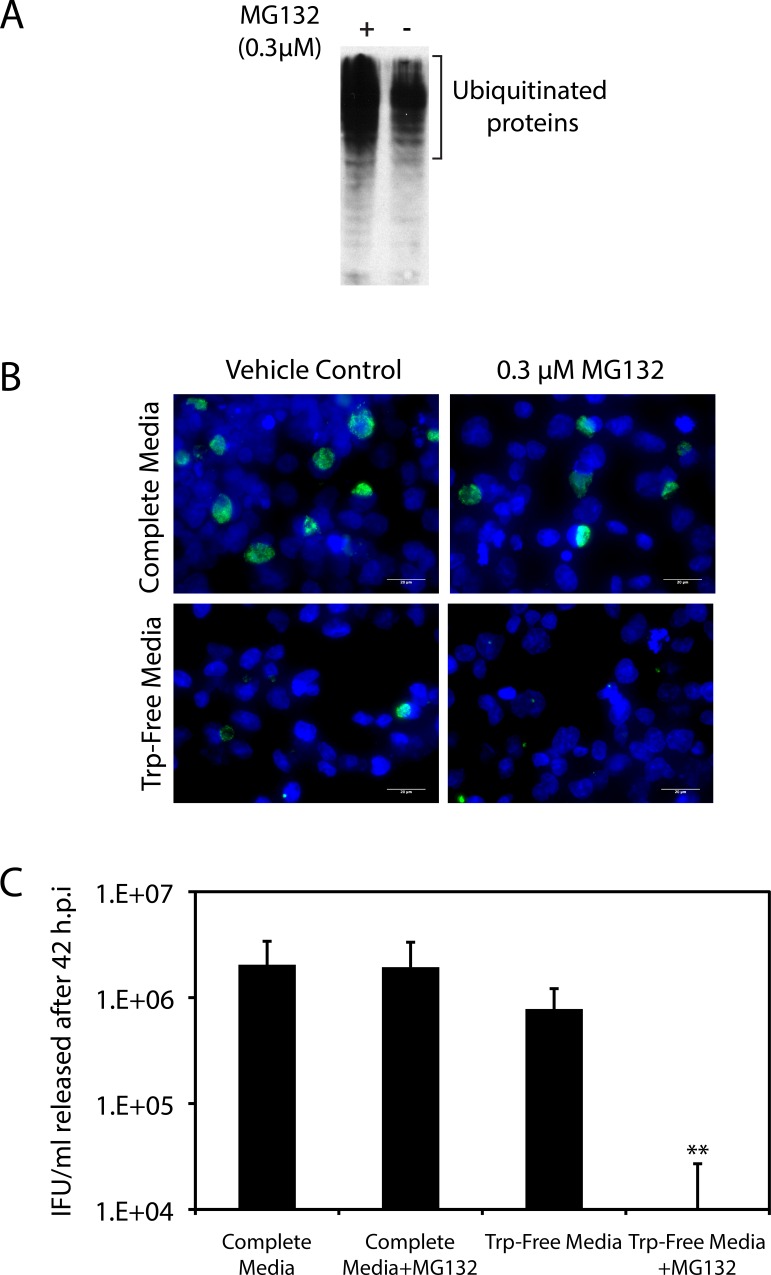
Proteasome activity is essential to support chlamydial development in C33A cells during tryptophan starvation. Immediately after infection with *C*. *trachomatis*, C33A cells were grown under four conditions: Complete media + vehicle control; Complete Media + 0.3 μM MG132; Trp-free media + vehicle control; and Trp-free media with 0.3 μM MG132. After 42 hours of growth under these conditions, inclusion development and IFU recovery was evaluated. A) Immunoblot evaluating the accumulation of polyubiquitinated proteins in C33A cells 24 hours post-exposure to the vehicle control or 0.3 μM MG132. The primary antibody used a rabbit polyclonal anti-ubiquitin antibody. B) Immunofluorescence using a FITC-conjugated anti-chlamydial LPS antibody was used to evaluate inclusion formation under the four conditions described above. Cells were counterstained with Hoechst dye. C) IFU/mL recovered 42 h.p.i. from cells grown under the four conditions described above. IFU recovery was quantified by infection of HeLa cells. The addition of 0.3 μM MG132 dramatically reduced IFU/mL released from C33A cells grown in Trp-Free Media (** indicates p < 0.001 by the Wilcoxon rank sum test). The bars represent the mean and standard deviation from three independent experiments.

An intricate panoply of intracellular sensors control amino acid homeostasis during nutrient starvation of mammalian cells [30, 32]. When any specific amino acid is depleted, there is an increase in the levels of uncharged tRNAs for that amino acid. The latter bind and activate the GCN2 kinase that phosphorylates eIF2α thereby blocking protein synthesis until nutrient availability is restored. Published experiments conducted using methionine-free and cysteine-free media have revealed proteasome activity to be critical for the maintenance of intracellular amino acid homeostasis during starvation [31]. Mammalian cells grown in media lacking cysteine or methionine survived for an extended time, with low levels of eIF2α phosphorylation, unless the proteasome was pharmacologically inhibited using MG132 [31]. Therefore, we tested the hypothesis that proteasome-dependent tryptophan salvage permitted chlamydial development in C33A cells grown in Trp-Free Media by examining the effect of MG132 on chlamydial development under these conditions. *C*. *trachomatis* infected cells were grown for 42 hours in Complete Media or Trp-Free Media and in the presence or absence of 0.3 μM MG132, and evaluated for inclusion development and IFU recovery (Fig 4). In control experiments, 0.3 μM MG132 was found to not impact C33A cell survival for up to 48 hours of exposure (data not shown). The capacity of 0.3 μM MG132 to block proteasome function was evaluated by examining the level of ubiquitinated proteins in C33A cell extracts after 24 hours of exposure to MG132. As shown in Fig 4A, MG132 increased the level of ubiquitinated proteins, consistent with proteasome inhibition. At this concentration, MG132 did not impact the size/number of inclusions, or IFU recovery, for infected cells grown in Complete Media (Fig 4B and 4C). In contrast, MG132 exposure resulted in a dramatic decrease in the size of inclusion and IFU recovery when infected cells were grown in Trp-Free Media. The reduction in IFU recovery in C33A cells by MG132 exposure during tryptophan starvation mirrored the reduction observed by tryptophan starvation alone in HeLa cells. Therefore we considered the possibility that an intrinsic difference in proteasome activity between HeLa and C33A cells may cause differing levels of free intracellular tryptophan during growth in Trp-Free Media, and consequently result in disparate patterns of chlamydial growth under these conditions.

### High levels of IFΝγ partially restores chlamydial development in HeLa cells

**Fig 5:**
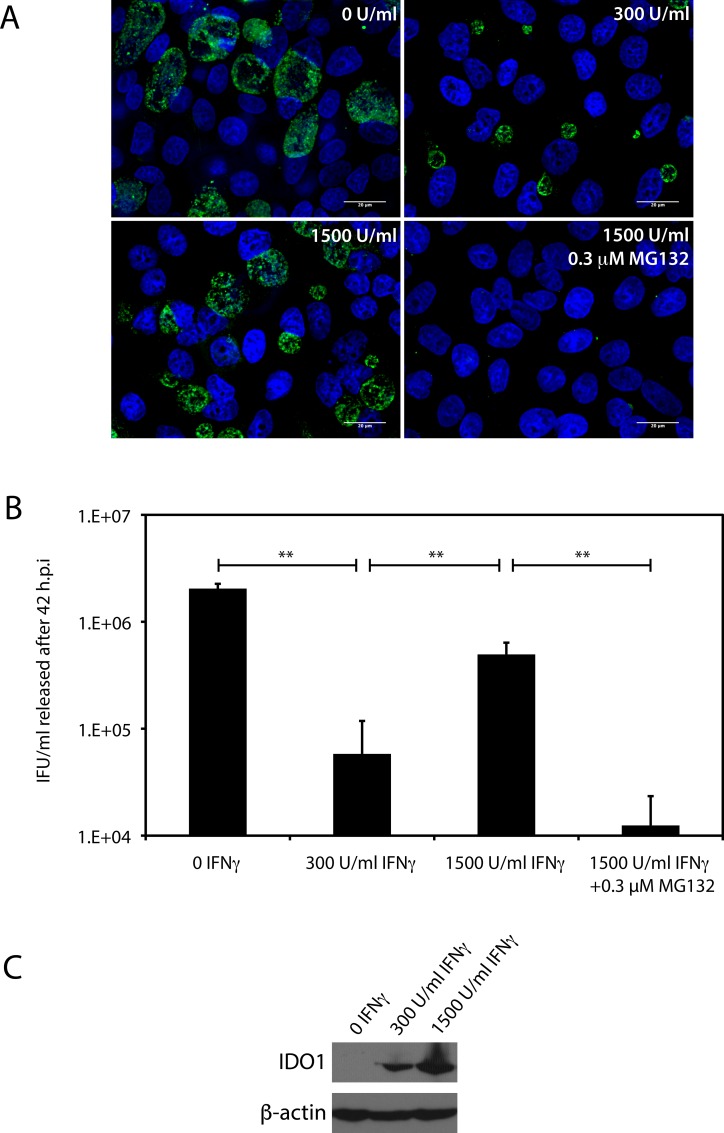
High levels of IFNγ partially restores *C*. *trachomatis* development in HeLa cells. After infection with *C*. *trachomatis*, HeLa cells were grown in DMEM containing 4 mg/L of tryptophan, and treated with 0, 300, 1500 U/mL of IFNγ, or I500 U/mL IFNγ + 0.3 μM MG132 for 42 hours, following which inclusion formation and IFU recovery were evaluated. A) Immunofluorescence using a FITC-conjugated anti-chlamydial LPS antibody was used to evaluate inclusion formation under the four conditions described above. Cells were counterstained with Hoechst dye. B) IFU/mL recovered at 42 h.p.i. from cells grown under the four conditions described above. IFU recovery was quantified by infection of HeLa cells. As reported previously [19], the addition of 300 U/mL IFNγ resulted in a statistically significant 2-log decrease in IFU recovery. Addition of 1500 U/mL IFNγ resulted in a statistically significant ~1-log increase in the IFU recovered relative to the addition of 300 U/mL IFNγ. IFU recovery was addition of 1500 U/mL IFNγ was significantly reduced by the concurrent administration of 0.3 μM MG132. The data represent three independent experiments (** indicates P < 0.001 by the Wilcoxon rank sum test).

The HR-HPV E6 and E7 oncogenes are known to reduce MHC Class I antigen presentation by altering the expression of several proteasome and immunoproteasome subunits [35–37]. These studies have also shown that addition of high levels of IFNγ (1500 U/mL) to HeLa and the HeLa sub-clone, HEp-2 cells, results in immunoproteasome formation, with the latter degrading a different subset of substrates relative to the constitutive proteasomes in these cells [37]. Multiple groups, including ours, have previously demonstrated that treatment of HeLa cells with 300 U/mL of IFNγ reduces *C*. *trachomatis* IFU release by ~2-logs [9, 11, 19]. While this level of IFNγ is sufficient to induce tryptophan depletion, it is insufficient to induce immunoproteasomes in HeLa cells [35–37], and consequently any possible intracellular tryptophan replenishment via immunoproteasome activity. If the activity of immunoproteasomes can increase intracellular free tryptophan levels, it is anticipated that exposure of infected cells to a high level of IFNγ (1500 U/mL) will be less restrictive to *C*. *trachomatis* growth than a lower level of IFNγ (300 U/mL). Therefore, we compared the effects of high and low levels of IFNγ on chlamydial inclusion development and IFU recovery within HeLa cells, with the results shown in Fig 5. As seen in Fig 5A, inclusions formed in the presence of 1500 U/mL of IFNγ were larger in size than those formed in the presence of 300 U/mL of IFNγ Moreover, several inclusions formed at 1500 U/mL of IFNγ resembled “normal” inclusions formed in the absence of IFNγ (*ibid*). Concomitant with the difference in inclusion phenotype, there was a significant increase in the IFU released from infected cells treated with 1500 U/mL of IFNγ relative to 300 U/mL of IFNγ (Fig 5B). This increase in chlamydial development is unlikely to result from a failure in IFNγ signaling at 1500 U/mL, because IDO1 expression was induced efficiently at this concentration (Fig 5C). The level of IFNγ we have used (1500 U/mL) has been demonstrated previously to induce immunoproteasomes in HeLa cells. To confirm that the restored chlamydial developed we observed under these conditions resulted from increased proteasomal activity, 0.3 μM MG132 was added concurrently with IFNγ (1500 U/mL), and found to completely abrogate inclusion development and IFU release (Fig 5A and 5B). We interpret these experiments to reiterate a role for proteasome-mediated tryptophan salvage in modulating the susceptibility of *C*. *trachomatis* to tryptophan starvation.

### The HR-HPV E6 oncogene accentuates the effect of tryptophan starvation on *C*. *trachomatis* development

**Fig 6:**
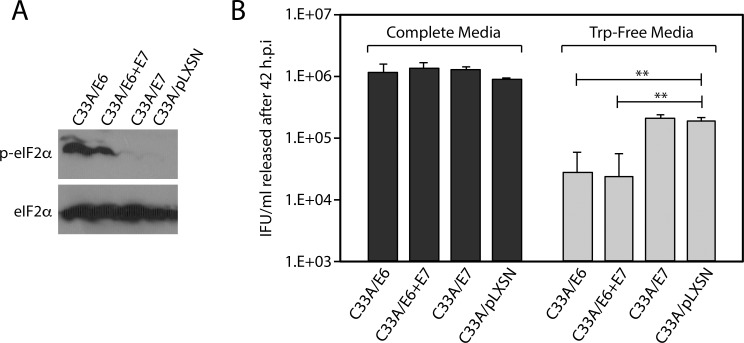
Expression of the HPV16 E6 oncogene in C33A cells accentuates the effect of tryptophan starvation on *C*. *trachomatis* development. Stable C33A-derivative cell-lines were constructed by transducing C33A cells with a control retroviral vector (pLXSN), or pLXSN derivatives expressing the HPV16 E6, E6 & E7, or E7 oncogenes. Stable cell-lines were selected using G418 as described in the material and method section. A) Cell-lines were grown in tryptophan-free media for 12 hours after which immunoblots were used to query the phosphorylation status of eIF2α. Total eIF2α was used as a loading control. B) IFU/mL recovered at 42 h.p.i. after infected cells were grown in complete media or tryptophan free-media, as evaluated by infection of HeLa cells. As anticipated, growth in tryptophan-free media reduced IFU/mL recovered from all four cell-lines. IFU/mL recovery from C33A/E6 and C33A/E6+E7 cell-lines during tryptophan starvation were significantly lower than the IFU/mL recovered from the control C33A/pLXSN cell-line. The data represent three independent experiments (** indicates P < 0.01 by the Wilcoxon rank sum test).

The data in Fig 1 indicates that chlamydial development and IFU release is restricted to a greater degree in HeLa and A2EN cells relative to C33A and 293 cells. These four lines have distinct origins; however, a commonality between HeLa and A2EN is that they express the E6 and E7 oncoproteins from HPV18 and HPV16 respectively [26]. C33A and 293 cells are similar in that they do not express any HPV oncogenes (Table 1). Because HR-HPV E6 and E7 oncogenes affect the expression of proteasome subunits and the choice of proteasomal targets, we tested whether the ectopic expression of the E6 and E7 oncogenes from HPV16 in C33A cells would phenotypically affect *C*. *trachomatis* growth in Trp-Free Media [36]. To do so, C33A cells were transduced with derivatives of the retroviral vector pLXSN that expressed HPV16 E6, E7, or both E6 & E7, following which stable cell-lines were obtained by G418 selection. Expression of E6 or E7 did not dramatically alter the growth of C33A cells in complete media, or survival in tryptophan-free media for 48 hours (data not shown). The level of free intracellular tryptophan was measured after the four cell-lines were grown in Trp-Free Media for 20 hours (Table 3). C33A/E6 and C33A/E6+E7 cells had reduced intracellular tryptophan levels relative to C33A/pLXSN and C33A/E7 cells; the levels in the latter two being comparable to those observed in parental C33A cells. Concomitant with this decrease, eIF2α phosphorylation was observed in C33A/E6 and C33A/E6+E7 cells by 12 hours post-withdrawal of tryptophan (Fig 6A), a marked difference from the experimental outcome in C33A/E7 and C33A/pLXSN cells (*ibid*). Next, we examined whether expression of E6 and/or E7 affected the outcome of tryptophan-starvation on *C*. *trachomatis* IFU release. As shown in Fig 6B, expression of E6 was sufficient to accentuate the effect of Trp-Free on *C*. *trachomatis* development as evaluated by IFU release. As we observed previously for HeLa and A2EN cells, there was a significant ~2-log decrease in IFU released from C33A/E6 and C33A/E6+E7 cells when grown in Trp-Free Media (*ibid*). In contrast, expression of HPV16 E7 did not alter the phenotype of C33A cells vis-à-vis chlamydial growth during tryptophan starvation (*ibid*). Thus, we conclude that during tryptophan starvation, ectopic expression of HPV16 E6 reduces the intracellular availability of free tryptophan within C33A cells, a reduction that correlates with a decrease in productive *C*. *trachomatis* replication under these conditions.

**Table 3:** Intracellular free tryptophan concentration in C33A-derivative cell-lines grown in Complete Media or after 20 hours of growth in Trp-Free Media.

Cell Line	Complete Media (Trp = 4mg/L)	Trp-Free Media (Trp = 80 μg/L)	Percent Decrease in Trp-Free Media
C33A/pLXSN	76.1 ± 20.7 nmoles^1^	34 ± 4.8 nmoles	55%
C33A/E6	54.9 ± 12.5 nmoles	16.6 ± 2.3 nmoles	70%
C33A/E6+E7	56.2 ± 5.5 nmoles	13.8 ± 3.8 nmoles	75%
C33A/E7	47.5 ± 11.6 nmoles	28.6 ± 10.4 nmoles	40%

^1^Tryptophan concentrations were evaluated using extracts from 10^8^ cells using RP-HPLC.

### Differences in the amino acid composition of the human proteome and the HR-HPV E6 degradome correlate with E6-dependent effects on tryptophan starvation

The experimental observations described above reveal that after tryptophan withdrawal, the intracellular levels of free tryptophan are lower in HeLa cells and C33A derivatives that express the HPV16 E6 oncoprotein (Tables 2 & 3). Pharmacological inhibition of proteasome-dependent amino acid salvage blocks chlamydial development in tryptophan-starved C33A cells (Fig 4), and 293 cells (data not shown). Finally, high levels of IFNγ, previously shown to induce immunoproteasomes in HeLa cells [35], whose substrates differ from those of constitutive proteasomes (*ibid*), permits chlamydial development despite the robust induction of IDO1 (Fig 5). Therefore, we wondered whether the HR-HPV E6 oncoprotein changes intracellular amino acid availability by skewing tryptophan-poor proteins for amino acid salvage by degradation, providing a mechanism underlying the reduced level of free intracellular tryptophan in E6-expressing cells during tryptophan starvation. HR-HPV E6 targets proteins for degradation by multiple mechanisms, including: 1) Acting as a scaffold to mediate the ubiquitination of specific proteins by the ubiquitin ligase, E6AP (also called UBE3A); 2) Increasing the activity of E6AP on its substrates by facilitating its trimerization; and 3) Promoting the degradation of some proteins by a largely unexplored ubiquitination-independent mechanism [38]. To explore this hypothesis, we compared the amino acid composition of the known human proteome (*H*. *sapiens* genome release GRCh38) to the set of proteins degraded in an E6 or E6AP dependent manner. The known proteome consists of 38133 unique accession numbers inferred from the sequence of all detected transcripts (GRCh38 accession numbers beginning with NP). For the E6/E6AP degradome proteins were included for which published data exists indicating their degradation in an E6 or E6AP-dependent manner, while excluding specific isoforms known not to be degraded. This compositional data is present within S1 Spreadsheet and S2 Spreadsheet, and a statistical comparison of their amino acid compositions is presented in Table 4. Although the number of proteins currently known to be targeted for degradation in an E6 or E6AP dependent manner is relatively small (n = 152), the amino acid composition of this set differs significantly for several amino acids from the human proteome as a whole. For example, the average representation of tryptophan in the E6/E6AP degradome is 41% lower than that observed for the proteome. This striking decrease is highly significant (P < 10^−15^ by the Wilcoxon rank sum test), and more than twice that observed for any other amino acid. Among the other hydrophobic amino acids, phenylalanine and leucine are also represented at distinctly lower levels in the degradome relative to the whole proteome. Thus, while unanticipated, proteins currently known to be degraded by E6/E6AP are under-represented in tryptophan; consequently, lowered levels of free intracellular tryptophan will prevail if such proteins constitute a substantial fraction of proteins degraded in HR-HPV E6-expressing cells during tryptophan starvation.

**Table 4:** Amino acid compositional differences in the human proteome versus the HR-HPV E6 & E6AP degradome.

Amino-Acid	Known *H*. *sapiens* proteome (GRCh38) (N = 38133)^1^	HR-HPV E6 + E6AP degradome (N = 152)^2^	Degradome/Proteome Ratio (Percent Change)	P(two-sided)^3^
ALA	7.094 ± 2.626	6.817 ± 2.618	0.961 (- 3.9%)	0.085
ARG	5.761 ± 2.258	5.972 ± 1.862	1.037 (+ 3.7%)	3x10^-3^
ASN	3.554 ± 1.536	3.638 ± 1.162	1.023 (+ 2.3%)	0.286
ASP	4.668 ± 1.727	5.329 ± 1.32	1.141 (+ 14.1%)	1.9x10^-8^
CYS	2.4 ± 2.15	2.07± 1.341	0.863 (- 13.7%)	0.135
GLN	4.645 ± 1.897	4.866 ± 1.709	1.048 (+ 4.8%)	0.193
GLU	6.958 ± 2.858	7.468 ± 1.917	1.073 (+ 7.3%)	1.3x10^-4^
GLY	6.608 ± 2.722	6.79 ± 2.114	1.028 (+ 2.8%)	0.135
HIS	2.607 ± 1.329	2.659 ± 0.9663	1.02 (+ 2%)	0.092
ILE	4.384 ± 1.941	4.403 ± 1.795	1.004 (+ 0.4%)	0.817
LEU	9.991 ± 2.958	8.713 ± 2.202	0.872 (- 12.8%)	9.7x10^-9^
LYS	5.818 ± 2.77	5.833 ± 2.028	1.003 (+ 0.3%)	0.479
MET	2.321 ± 1.066	2.445 ± 1.039	1.053 (+ 5.3%)	0.102
**PHE**	**3.775 ± 1.697**	**3.049 ± 1.222**	**0.808 (-19.2%)**	**3.4x10**^**-10**^
PRO	6.139 ± 3.001	7.084 ± 2.725	1.154 (+ 15.4%)	2.3x10^-7^
SER	7.993 ± 2.596	8.283 ± 2.298	1.036 (+ 3.6%)	1.4x10^-2^
THR	5.209 ± 1.671	4.844 ± 1.107	0.93 (- 7%)	3.3x10^-3^
**TRP**	**1.278 ± 0.903**	**0.7545 ± 0.5107**	**0.590 (- 41%)**	**1.2x10**^**-16**^
TYR	2.776 ± 1.406	2.751 ± 0.9715	0.991 (- 0.9%)	0.422
VAL	6.02 ± 1.887	5.757 ± 1.934	0.956 (- 4.4%)	1x10^-2^

^1^The composition of individual proteins in the known proteome can be found in S1 Spreadsheet.

^2^The composition of individual proteins in the HR-HPV E6 & E6AP degradome can be found in S2 Spreadsheet.

^3^P(two-sided) was calculated using the Wilcoxon rank sum test.

**Fig 7:**
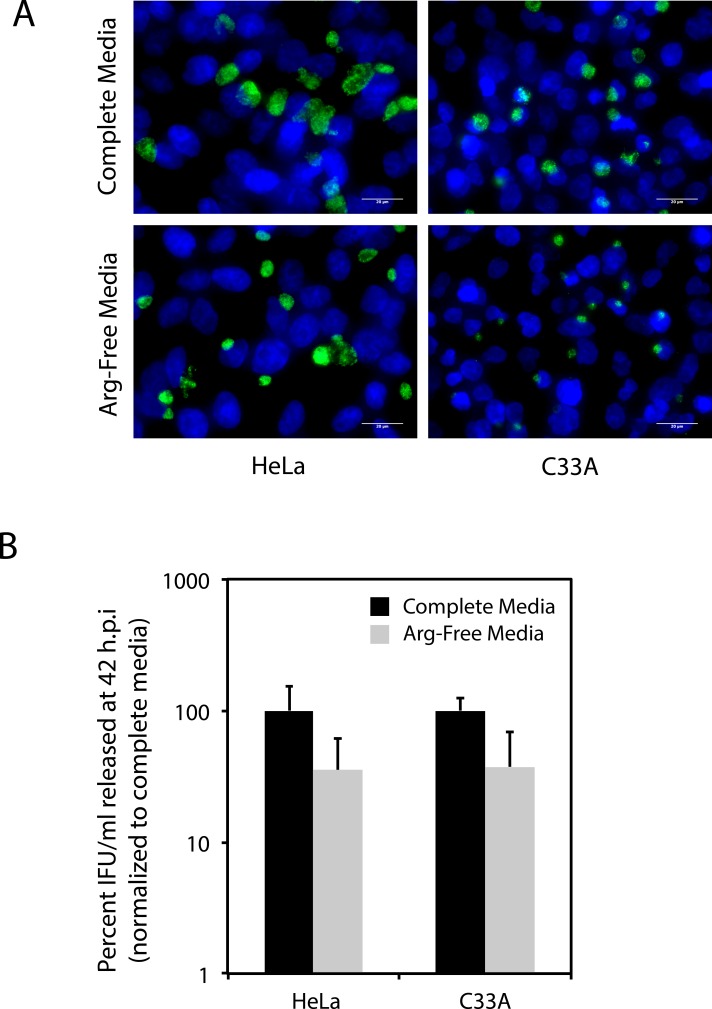
Arginine starvation impacts *C*. *trachomatis* development in HeLa and C33A cells to an equivalent extent. *C*. *trachomatis* infected HeLa and C33A cells were grown in Complete Media and arginine-free (Arg-Free) media for 42 h.p.i. at which inclusion development and IFU recovery were evaluated. A) Immunofluorescence using a FITC-conjugated anti-chlamydial LPS antibody was used to evaluate inclusion formation in HeLa and C33A cells grown in Complete Media or Arg-Free Media. Cells were counterstained with Hoechst dye. B) IFU/mL recovered at 42 h.p.i. after infected cells were grown in complete media or arginine free-media, as evaluated by infection of HeLa cells. Although the IFU/mL recovered under arginine starvation conditions was lower than that observed in complete media, the decrease was not observed to be statistically significant for either HeLa or C33A cells (P > 0.05 by the Wilcoxon rank sum test).

**Fig 8:**
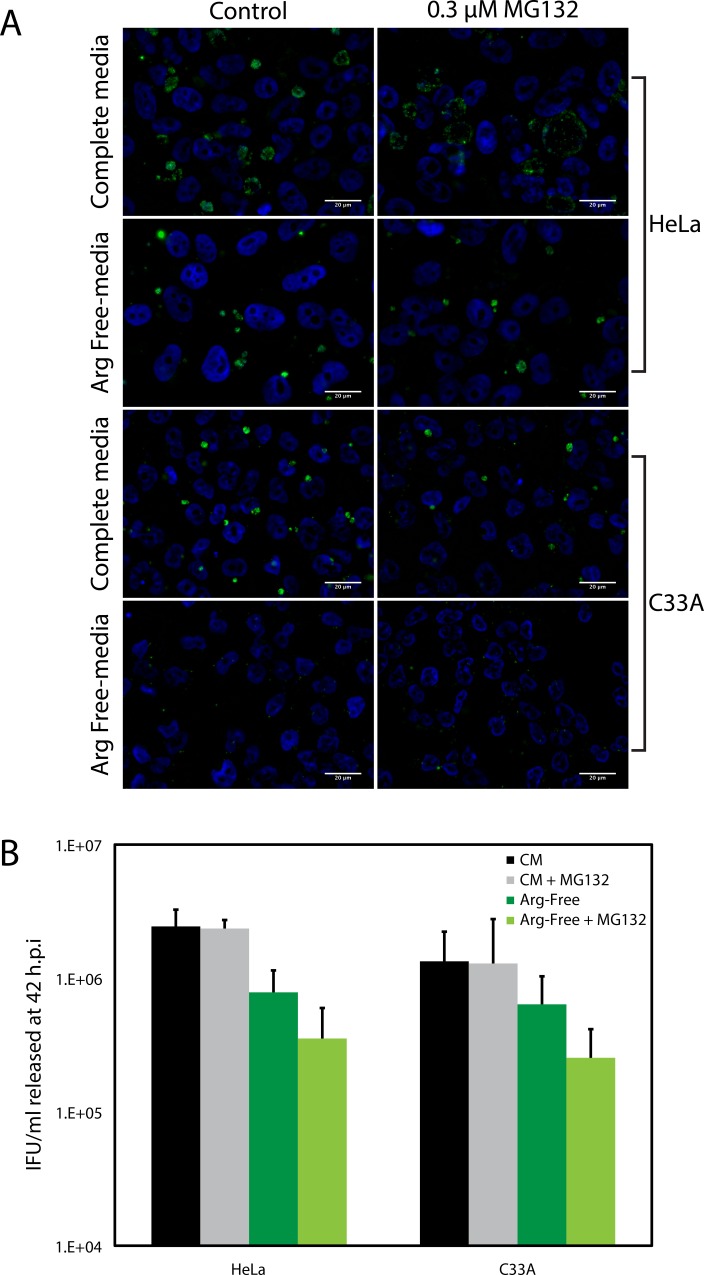
Proteasome inhibition equivalently affects *C*. *trachomatis* growth in HeLa and C33A during arginine starvation. *C*. *trachomatis* infected HeLa and C33A cells were grown in complete media (CM) or arginine free media (Arg-Free) with a vehicle control (CM, Arg-Free) or in the presence of 0.3 μM MG132 (CM + MG132, Arg-Free + MG132) for 42 h.p.i. At this time, cells were either stained to observe inclusion size or harvested to quantify IFU released. A) Immunofluorescence using a FITC-conjugated anti-chlamydial LPS antibody was used to evaluate inclusion formation in HeLa and C33A cells grown in CM, CM + MG132, Arg-Free, or Arg-Free + MG132. Cells were counterstained with Hoechst dye. B) IFU/mL recovered at 42 h.p.i. after infected cells were grown under the conditions used in “A”. IFU/mL was quantified by infection of HeLa cells. Although the IFU/mL recovered during arginine starvation was lower than that observed in complete media, the decrease was not observed to be statistically significant for either HeLa or C33A cells (P > 0.05 by the Wilcoxon rank sum test). The addition of MG132 did not affect IFU release for cells grown in CM. A statistically significant decrease (P < 0.05) was observed for cells grown in Arg-Free media.

The E6 proteins of several HPVs also interact with multiple proteasomal subunits, rendering it possible that the lower levels of free intracellular tryptophan detected (Tables 2 & 3) result from an overall diminished level of proteasome activity, rather than selection for a subset of proteins to be degraded [36]. To test this, we compared the effect of arginine starvation on *C*. *trachomatis* development in HeLa and C33A cells, because *C*. *trachomatis* is also an arginine auxotroph [39, 40]. Mammalian cells are conditionally auxotrophic for arginine, with the capacity to synthesize arginine from citrulline if the latter is provided in media [41]. When grown in complete media, the intracellular arginine concentration in HeLa cells was ~2 mM [42]. This concentration was reduced to 0.04 mM when cells were grown in media containing < 0.02 mM arginine [42]. Under both conditions, the intracellular concentration of citrulline remained lower than the limit of detection (< 0.001 mM), rendering citrulline an unlikely source for the 0.04 mM free intracellular arginine detected during arginine starvation (*ibid*). Thus, arginine salvage by protein degradation is most likely to be the mechanism by which intracellular arginine stores are maintained during arginine starvation. Unlike tryptophan, arginine is represented in the E6/E6AP degradome at approximately the same level as in the whole proteome (Table 4). Therefore, if there is no overall impairment to protein degradation in HR-HPV E6-expressing cells, it is anticipated that arginine starvation would affect *C*. *trachomatis* development in HeLa and C33A cells equivalently. As shown in Fig 7, equivalent numbers of normal looking inclusions were observed within C33A and HeLa cells infected with *C*. *trachomatis* during arginine starvation. Further, an equivalent decrease in IFU release was observed for both cell-lines when Arg-Free Media was compared to Complete Media. The outcome of this experiment better support the idea that the reduced intracellular levels of free tryptophan detected in HR HPV E6-expressing cells reflects the composition of proteins degraded in these cells, rather than a lower rate of protein degradation. Were the latter to be true, then Arg-free media would have impacted *C*. *trachomatis* growth in HeLa cells to a greater extent than C33A cells. To confirm this interpretation, we examined the effect of the proteasome inhibitor MG132 on *C*. *trachomatis* replication in HeLa and C33A cells grown in Arg-free media (Fig 8). The outcome of this experiment clearly demonstrated that MG132 equivalently affected *C*. *trachomatis* growth in both C33A and HeLa cells grown under these conditions. Because MG132 affects proteasome function, but not host protein synthesis, these results reiterate a key role for the proteasome in determining the free amino-acid pool.

## Discussion

A large body of evidence indicates IFNγ to be a major host protective cytokine response against genital *C*. *trachomatis* infections, where it functions by inducing the catabolism of tryptophan, an essential amino acid for *C*. *trachomatis*. Yet, the literature indicates cell-line dependent effects of tryptophan deprivation on the growth of *C*. *trachomatis*. In early studies, Karayiannis and Hobson found tryptophan-free media to reduce inclusion formation by a genital serovar of *C*. *trachomatis* in McCoy cells by ~4-fold, with a one-log decrease in yield of infectious units [43]. In contrast, later studies conducted using HeLa 229 cells indicate that tryptophan-free media reduces the infectious yield of *C*. *trachomatis* by 4–6 logs [14, 44]. While it is possible this disparity results from differences in the basal medium used, it is also possible that they reflect cell-line intrinsic differences in tryptophan availability during starvation. In the studies reported here, we have identified expression of the HR-HPV E6 oncogene as a key factor that modulates the effect of tryptophan-deprivation on chlamydial growth.

The intracellular pathways that compensate for amino acid starvation in mammalian cells are well understood [31, 45, 46]. When starvation ensues, the proteasome is used to maintain the intracellular free amino acid pool by salvaging amino acids, a process that is sufficient to maintain cell viability [31]. If the proteasome is inhibited, the consequential reduction in the size of the intracellular amino acid pool results in the phosphorylation of eIF2α, which in turns reduces amino acid consumption by blocking protein translation [32]. When *C*. *trachomatis*-infected cells are starved for tryptophan, bacteria and the host compete for this essential amino acid that both need for protein translation [47]. Thus, it is not surprising that cycloheximide alleviates the effect of tryptophan deprivation on the growth *C*. *trachomatis* in HeLa cells (S2A Fig) by blocking host protein synthesis. Similarly, growing C33A cells for 24 hours in Trp-Free Media prior to infection also blocks chlamydial growth, because the intracellular tryptophan has been consumed by the cells (S2B Fig). While these results render it possible that cell-line specific effects of tryptophan starvation result from differences in the host rate of tryptophan consumption, the possibility that the starting intracellular tryptophan pool differs between cell-lines during tryptophan starvation had not been considered previously. That difference, its effects on chlamydial biology, and the factors that contribute to it were the focus of this study.

During tryptophan starvation, chlamydial growth was found to be far more restricted in HeLa and A2EN cells, both of which express the HR-HPV E6 and E7 oncogenes, relative to C33A and 293 cells. Comparisons of the intracellular free tryptophan levels during starvation indicate the level in HeLa cells to be approximately 50% that found in C33A cells. It is unlikely that this difference results from differences in the rate of tryptophan consumption in these cells because both cell-lines double at approximately the same rate (18–22 hours) [48], and treatment with cycloheximide results in the same relative increase in *C*. *trachomatis* IFUs released in Trp-Free Media. Were HeLa cells to consume tryptophan at a higher rate than C33A cells, we would anticipate that the addition of cycloheximide would increase *C*. *trachomatis* replication to a greater extent in HeLa cells. The rates of eIF2α phosphorylation would also suggest that tryptophan starvation induces a block to protein synthesis in HeLa cells more rapidly than C33A cells [31]. The rapidity with which we have observed amino acid starvation to induce eIF2α phosphorylation in HeLa cells is not unique to tryptophan [32]; high baseline levels of eIF2α phosphorylation have been observed in HeLa cells, but not other cell-lines such as 293 (*ibid*). Further, p-eIF2α was seen to increase in HeLa cells within 30 minutes of exposure to leucine-free media and reaches maximal levels within 4–6 hours under these conditions (*ibid*).

The size of the free amino acid pool during starvation is determined by the rate of consumption and the rate at which amino acid salvage occurs [31, 32]. Our results indicate that the rates and/or nature of proteasome-dependent amino acid salvage differ between cell-lines. MG132-treatment reduces the IFUs recovered from *C*. *trachomatis*-infected C33A cells grown in Trp-Free Media by ~2-logs, a decrease that parallels the decrease observed in HeLa cells when Trp-Free Media is used by itself. This conclusion is reiterated by studies in which *C*. *trachomatis*-infected HeLa cells were exposed to high levels of IFNγ previously shown to induce immunoproteasomes [49]. An approximately 2-log decrease in IFU released was observed when infected HeLa cells were exposed to a moderate concentration of IFNγ (300 U/mL). Paradoxically, high levels of IFNγ (1500 U/mL) had a smaller effect on IFU release. Co-administration of 1500 U/mL IFNγ along with MG132 strongly inhibited IFU release, corroborating previous reports that these levels of IFNγ induce the formation of active immunoproteasomes in HeLa cells, and reiterating a role for proteasomes in amino acid salvage during starvation.

Immunoproteasomes in HeLa cells are reported to have a different set of substrates than the constitutive proteasomes in these cells [35, 37, 50, 51]. This observation prompted us to consider whether cell-line specific differences in the pool of substrates by actively processed by the proteasome could affect the size and/or composition of the free intracellular tryptophan pool. It is known that the HR-HPV E6 and E7 oncogenes affect proteasome subunit expression, interact with proteasome subunits, and have been reported to reduce MHC Class I antigen presentation by modulating proteasome activity [35–37]. Therefore, we considered the possibility that the expression of HPV16 E6 & E7 in HPV-negative cells might reduce overall proteasome activity to decrease the size of the free amino acid pool. We tested this hypothesis by constructing C33A derivative cell-lines that expressed HPV16 E6, E7, or both, and then evaluated their capacity to support *C*. *trachomatis* development and IFU release in Trp-Free Media. The outcome of this experiment was intriguing; although both E6 and E7 have been reported to influence the expression of proteasome subunits, expression of E6 alone reduced *C*. *trachomatis* IFU release by ~1.5 logs during growth in Trp-Free Media. Further, this decrease correlated with a reduction in free intracellular tryptophan levels during growth in Trp-Free Media. The mechanism by which HPV16 E6 induces this difference is unclear, but may reflect the composition of the host proteins it targets for degradation. Besides it well-described substrate, p53, HR-HPV E6 induces the degradation of a large number of cellular proteins whose functions include: 1) Epithelial cell polarity; 2) Formation and maintenance of cell junctions; 3) Modulation of telomerase transcription; 4) Pro-inflammatory processes driven by cytokines such as IL-1β; and 5) Modulation of protein phosphorylation status. Most of these proteins are degraded because E6 facilitates their ubiquitination by the ubiquitin ligase E6AP, also called UBE3A [52]. HR-HPV E6 also dramatically increases the specific activity of E6AP by promoting its trimerization [38]; therefore, while expression of HPV16 E6 doesn’t dramatically change the E6AP interactome, biochemical data are consistent with the interpretation that there is increased turnover of proteins normally targeted by E6AP for degradation (*ibid*). While the list of proteins targeted by E6/E6AP is not large, we evaluated whether the amino acid composition of this degradome differed from the known human proteome. Surprisingly, it does; the composition of proteins in the degradome reveals them to be severely and significantly under-represented in tryptophan (41% lower), phenylalanine (19% lower) and leucine (12% lower) relative to the human proteome (Table 2; S3 E6+E6AP_DegradomeComposition.xlsx). In this context, it is of interest that leucine starvation affects HeLa cells more profoundly than valine starvation (4.4% lower in the degradome), or methionine starvation (5.3% greater in the degradome) [53–55]. These studies also reveal that methionine deprivation has a minor effect on protein synthesis in HeLa cells, with intracellular protein turnover used as the source for methionine when it is omitted from media (*ibid*). Our protein compositional analysis revealed arginine representation in the degradome to be similar to the entire proteome (3.7% greater in the degradome), suggesting that the effect of arginine starvation on *C*. *trachomatis* replication would be similar in HeLa and C33A cells. We tested this hypothesis by evaluating the effect of arginine starvation on chlamydial development and IFU release in HeLa and C33A cells, and as predicted found it to be similar in both cell lines (Fig 7). We note that proteasome inhibition studies better support the hypothesis that differences in the free intracellular amino-acid pool between HeLa and C33A cells result from differences in the set of proteins being recycled by proteolysis, rather than an overall lower rate of protein turnover in HeLa cells.

Regardless of the mechanism by which it acts, by reducing the size of the intracellular free tryptophan pool, HR-HPV E6 influences the outcome of tryptophan starvation on *C*. *trachomatis*. During starvation, *C*. *trachomatis* continues to develop in cell-lines that lack E6; thus, during tryptophan starvation, such as that induced by IFNγ, doxycycline is more effective against *C*. *trachomatis* infections of cell-lines with larger tryptophan stores. Given their overlapping tissue tropism, it is possible that infection of a rare HR-HPV positive cell can protect *C*. *trachomatis* against antibiotic-mediated clearance, especially during a robust IFNγ-dependent protective immune response [56–59]. A recent meta-analysis study including 194 studies consisting of 1,016,719 women with normal cytological finding estimated global prevalence of HPV to be 11.7%, with the peak age of HPV distribution at <25 years, same as that for *C*. *trachomatis* [60]. These undetected and unappreciated HPV infections provide proliferating cells that express E6 oncogene that can modulate *C*. *trachomatis* development. Another study showed 20% of HPV positive biopsies containing non-neoplastic epithelium were stained positive for *C*. *trachomatis* [61]. Likewise, a cross-sectional study done in indigenous women from Paraguay showed 21.4% of HPV positive women were also positive with *C*. *trachomatis* and the frequency of *C*. *trachomatis* infection in HPV positive women was statistically significant [62]. In fact, *C*. *trachomatis* was the only other genital infection whose co-infection with HPV was statistically significant (*ibid*). This can either mean the risk factors that are associated with HPV also cause *C*. *trachomatis* infections, or alternatively, the presence of HPV provides favorable conditions for *C*. *trachomatis* infections, or survival of *C*. *trachomatis*.

We speculate that co-infection of an HPV-positive cell can promote *C*. *trachomatis* survival in two ways. Approximately 20% of infected women spontaneously clear *C*. *trachomatis* infections, most likely by inducing an IFNγ response. During such a response, we anticipate that tryptophan levels will be lower in HPV-positive cells relative to HPV-negative cells, further reducing *C*. *trachomatis* gene expression, and ergo the synthesis of bacterial antigens targeted by the protective host T-cell response. As a consequence, *C*. *trachomatis* could potentially enter an antigenically silent state within such cells promoting its survival until the host immune response wanes. Alternatively, reduced tryptophan levels within HPV-positive co-infected cells may decrease bacterial translation during an IFNγ response, and reduce the efficacy of antibiotics against *C*. *trachomatis*. Of note, there is evidence of recurrent cervical chlamydial infections even in the absence of reinfection, and despite antibiotic administration [63].

It is also clear that HPV and *C*. *trachomatis* can infect the same tissue, rendering rare co-infections possible. In a study to detect HPV DNA in cancerous tissue, HPV DNA was detected in 91% mucinous adenocarcinoma, which includes, endocervical, intestinal, and endometrioid histological subtypes and 100% in adenosquamous tumors [64]. This high prevalence of HPV DNA is comparable to the prevalence of HPV in cervical squamous cell carcinoma. Because these cancers arise from the rare transformation of an HPV-infected cell, it is extremely likely that larger numbers phenotypically normal HPV-infected cells are in tissues commonly associated with genital *C*. *trachomatis* infections; the cells will not be detected by cytological procedures that rely on cell phenotype. Indeed, several recent studies have shown that between 7–11% of cervical samples that show normal cytology are positive for high-risk HPV by other techniques such as PCR [65–68].

While we have focused on differences in protein degradome composition between cell-lines as a determinant *C*. *trachomatis* susceptibility to tryptophan starvation, it is important to note that there are multiple factors that can affect the tryptophan pool during IFNγ exposure, including the level to which IDO1 is induced [11, 69], and the rates of host protein synthesis. Further, it is known that different serovars of *C*. *trachomatis* differ dramatically in their susceptibility to IFNγ exposure [70]. This may also occur for a multitude of reasons, including a serovar-specific effect on host pathways required to induce IDO1 or possibly rate of host translation. We also note that *C*. *trachomatis* secretes bacterial proteases into the host cell as reviewed in [71]; in addition to targeting specific host proteins whose degradation is necessary for bacterial replication, the outcome of their activity is also predicted to alter the cytoplasmic free amino-acid pool. Thus, serovar-specific differences in the level of expression or activity of these bacterial enzymes will also impact the effect of amino-acid starvation on *C*. *trachomatis*.

The majority of cell-lines used to conduct studies with *C*. *trachomatis* and other intracellular pathogens have been transformed using viral oncogenes, including HR-HPV E6 & E7 [72, 73]. We did not anticipate HR-HPV E6 to influence the effect of tryptophan starvation on *C*. *trachomatis*, and were surprised to observe the effect described in this report. Because these viral oncogenes affect a multitude of cellular processes, our results render it desirable to evaluate the biology of intracellular pathogens in primary cells or non-virally immortalized cell-lines.

## Supporting Information

S1 Fig Single-step growth curve for *C*. *trachomatis* in HeLa and C33A cell lines grown in Complete Media and Trp-Free Media. HeLa and C33A cells were infected with *C*. *trachomatis* at an m.o.i of 1 and grown in Complete Media or Trp-Free Media. Infected cells were harvested at the indicated times point infection, and extracts were used to quantify IFU/mL. The data represents the results obtained from three independent experiments.
(TIF)

S2 Fig *C*. *trachomatis* growth in presence of cycloheximide or in cells starved for tryptophan 24 hours prior to infection. A) Cycloheximide treatment rescues the effect of Tryptophan-Free media on *C*. *trachomatis* replication in HeLa cells. After infection with *C*. *trachomatis* (m.o.i 5), HeLa and C33A cells were incubated in the indicated media containing cycloheximide (CHX). After 42 h.p.i cells were harvested and IFU/mL was quantified as described in experimental procedures section. Data is shown as log % with the IFU/ml Released in Complete Media + CHX for each cell line set to 100%. B) Exposure of C33A cells to Tryptophan-Free Media for 24 hours prior to infection reduces *C*. *trachomatis* replication. C33A cells were grown in Trp-Free Media for 24 hours, after which they were infected with *C*. *trachomatis* (m.o.i 5) and grown in Complete Media or Trp-Free Media. IFU/mL recovered at 42 h.p.i. was evaluated as described in the experimental procedures section. Data is shown as log % with the Complete Media values for each cell line set to 100%. The data represents results obtained from three independent experiments.
(TIF)

S1 Spreadsheet Hs_ProteomeComposition.xlsx. A Microsoft Excel spreadsheet containing the amino acid composition of all known proteins present in the human genome release GRCh37.
(XLSX)

S2 Spreadsheet E6+E6AP_DegradomeComposition.xlsx. A Microsoft Excel spreadsheet containing the amino acid composition of all proteins currently known to be degraded in a manner dependent on high-risk HPV E6 and/or the ubiquitin ligase E6AP. Citations are included for each protein as a PMID adjacent to the accession number for that protein.
(XLSX)

S1 Text AAComposition.sh. A *bash* shell script used to determine the amino acid composition of a set of proteins, each of which has to be in FASTA format file. The output from this script is tab-delimited and compatible with Microsoft Excel.
(SH) Click here for additional data file.
