# Supplementary figures and images for: The High-Risk Human Papillomavirus E6 Oncogene Exacerbates the Negative Effect of Tryptophan Starvation on the Development of Chlamydia trachomatis
Source: PLoS One. 2016 Sep 22;11(9):e0163174. doi: 10.1371/journal.pone.0163174 (PMC5033384; doi:10.1371/journal.pone.0163174)

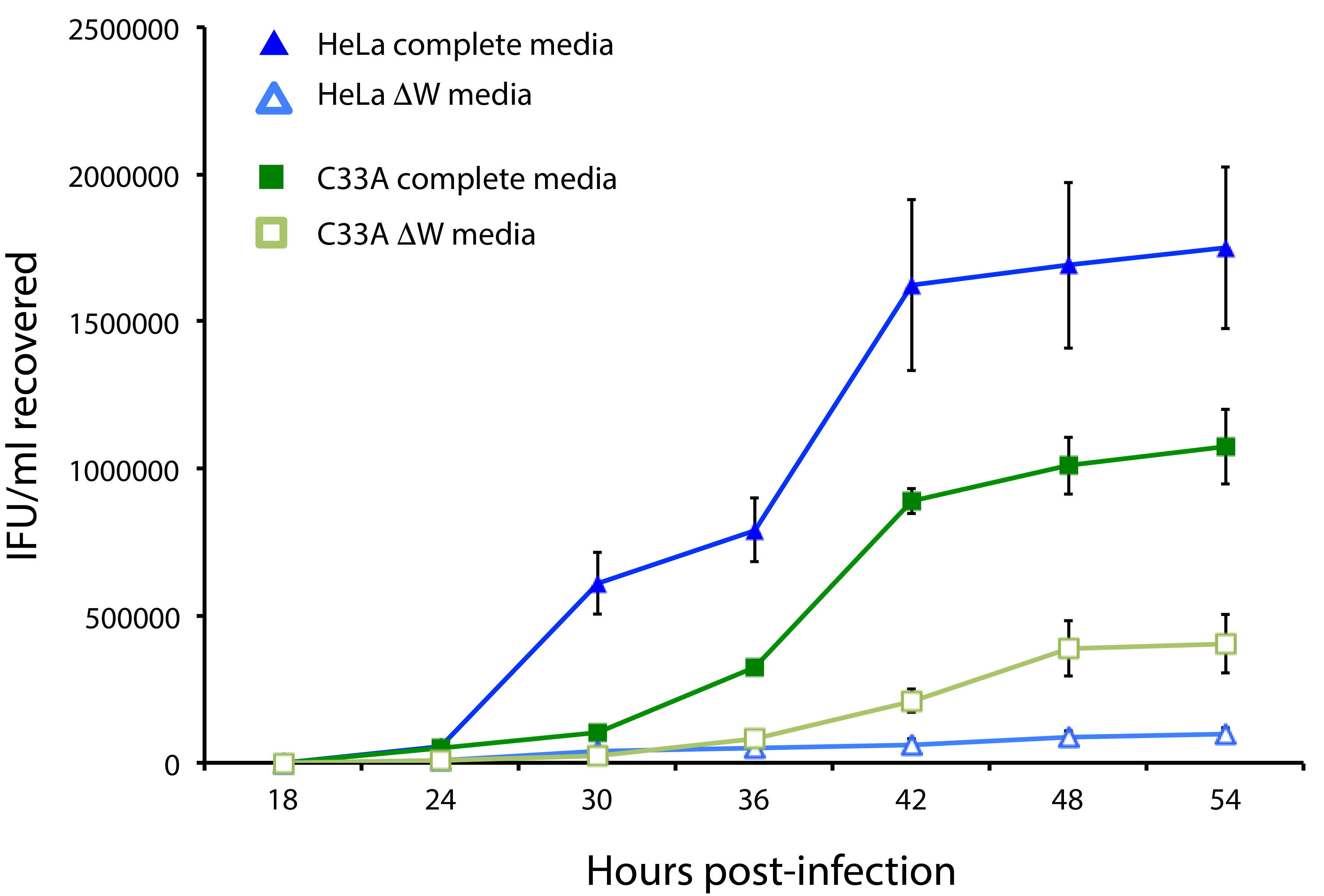

Supplement: S1 Fig — HeLa and C33A cells were infected with C. trachomatis at an m.o.i of 1 and grown in Complete Media or Trp-Free Media. Infected cells were harvested at the indicated times point infection, and extracts were used to quantify IFU/mL. The data represents the results obtained from three independent experiments. (TIF) [file pone.0163174.s001.tif]

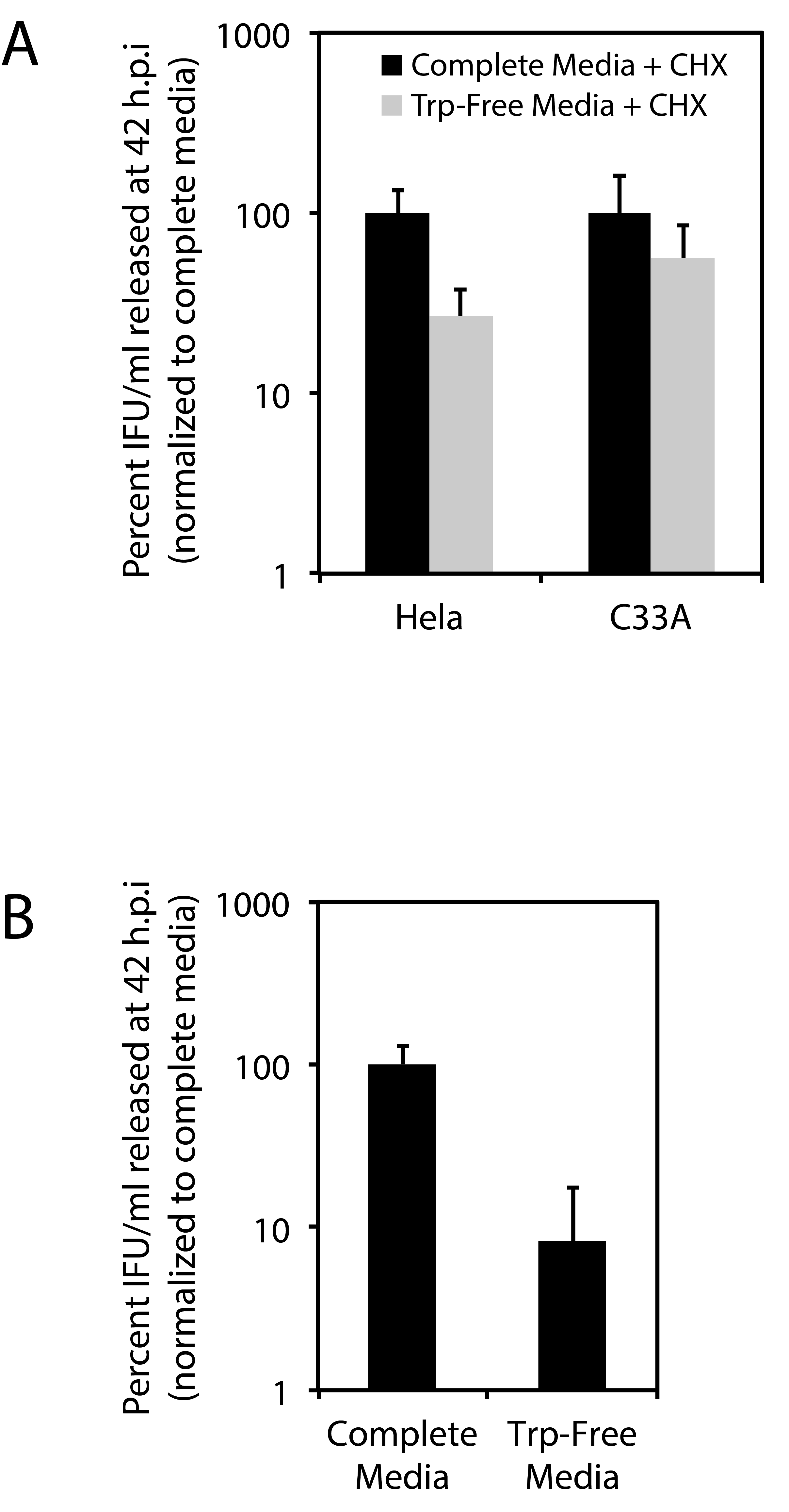

Supplement: S2 Fig — A) Cycloheximide treatment rescues the effect of Tryptophan-Free media on C. trachomatis replication in HeLa cells. After infection with C. trachomatis (m.o.i 5), HeLa and C33A cells were incubated in the indicated media containing cycloheximide (CHX). After 42 h.p.i cells were harvested and IFU/mL was quantified as described in experimental procedures section. Data is shown as log % with the IFU/ml Released in Complete Media + CHX for each cell line set to 100%. B) Exposure of C33A cells to Tryptophan-Free Media for 24 hours prior to infection reduces C. trachomatis replication. C33A cells were grown in Trp-Free Media for 24 hours, after which they were infected with C. trachomatis (m.o.i 5) and grown in Complete Media or Trp-Free Media. IFU/mL recovered at 42 h.p.i. was evaluated as described in the experimental procedures section. Data is shown as log % with the Complete Media values for each cell line set to 100%. The data represents results obtained from three independent experiments. (TIF) [file pone.0163174.s002.tif]
